# Supplementary material for: Fungal melanin suppresses airway epithelial chemokine secretion through blockade of calcium fluxing
Source: Nat Commun. 2024 Jul 10;15:5817. doi: 10.1038/s41467-024-50100-x (PMC11237042; doi:10.1038/s41467-024-50100-x)
Supplement: Supplementary file 3 — Reporting Summary [file 41467_2024_50100_MOESM3_ESM.pdf]

nature portfolio

Corresponding author(s): Jatin M Vyas

Last updated by author(s): Jun 17, 2024

Reporting Summary

Nature Portfolio wishes to improve the reproducibility of the work that we publish. This form provides structure for consistency and transparency in reporting. For further information on Nature Portfolio policies, see our Editorial Policies and the Editorial Policy Checklist.

Statistics

For all statistical analyses, confirm that the following items are present in the figure legend, table legend, main text, or Methods section.

n/a	Confirmed	
☐	☒	The exact sample size (*n*) for each experimental group/condition, given as a discrete number and unit of measurement
☐	☒	A statement on whether measurements were taken from distinct samples or whether the same sample was measured repeatedly
☐	☒	The statistical test(s) used AND whether they are one- or two-sided<br>*Only common tests should be described solely by name; describe more complex techniques in the Methods section.*
☒	☐	A description of all covariates tested
☒	☐	A description of any assumptions or corrections, such as tests of normality and adjustment for multiple comparisons
☐	☒	A full description of the statistical parameters including central tendency (e.g. means) or other basic estimates (e.g. regression coefficient) AND variation (e.g. standard deviation) or associated estimates of uncertainty (e.g. confidence intervals)
☐	☒	For null hypothesis testing, the test statistic (e.g. *F*, *t*, *r*) with confidence intervals, effect sizes, degrees of freedom and *P* value noted<br>*Give P values as exact values whenever suitable.*
☒	☐	For Bayesian analysis, information on the choice of priors and Markov chain Monte Carlo settings
☒	☐	For hierarchical and complex designs, identification of the appropriate level for tests and full reporting of outcomes
☒	☐	Estimates of effect sizes (e.g. Cohen's *d*, Pearson's *r*), indicating how they were calculated

Our web collection on statistics for biologists contains articles on many of the points above.

Software and code

Policy information about availability of computer code

Data collection	N/A
Data analysis	GraphPad Prism v10.2.3, LEGENDplex Data Software Suite, FlowJo 10 software, Adobe Photoshop 2021, MetaMorph software v7.10.5.476, Adobe Illustrator vCS4, ImageJ v 1.54f

For manuscripts utilizing custom algorithms or software that are central to the research but not yet described in published literature, software must be made available to editors and reviewers. We strongly encourage code deposition in a community repository (e.g. GitHub). See the Nature Portfolio guidelines for submitting code & software for further information.

Data

Policy information about availability of data

All manuscripts must include a data availability statement. This statement should provide the following information, where applicable:

- Accession codes, unique identifiers, or web links for publicly available datasets
- A description of any restrictions on data availability
- For clinical datasets or third party data, please ensure that the statement adheres to our policy

The datasets generated during and/or analyzed during the current study are available in the source data supplemental file submitted with the manuscript.

Research involving human participants, their data, or biological material

Policy information about studies with human participants or human data. See also policy information about sex, gender (identity/presentation), and sexual orientation and race, ethnicity and racism.

Reporting on sex and gender	Both males and females without known lung disease were used as a source of human basal cells, since donors were anonymous to the investigator, biological sex or gender was not considered.
Reporting on race, ethnicity, or other socially relevant groupings	Not collected
Population characteristics	No known history of lung disease
Recruitment	Consent was provided for tissue analysis as per approved protocol. Adult participants were recruited regardless of sex, gender, age, or ancestry. Per approved IRB protocol, no demographic information was collected for blood samples and thus, no information was provided for these.
Ethics oversight	Approved by MGH IRB Protocol numbers 2022P002626 and 2015P000818.

Note that full information on the approval of the study protocol must also be provided in the manuscript.

Field-specific reporting

Please select the one below that is the best fit for your research. If you are not sure, read the appropriate sections before making your selection.

☒ Life sciences ☐ Behavioural & social sciences ☐ Ecological, evolutionary & environmental sciences

For a reference copy of the document with all sections, see nature.com/documents/nr-reporting-summary-flat.pdf

Life sciences study design

All studies must disclose on these points even when the disclosure is negative.

Sample size	For all in vitro experiments, we used a minimum n=3 replicates based on prior experiment. Thus, no power calculations were conducted for these studies. For in vivo neutrophil recruitment, a sample size of n=5 mice per experimental condition based on prior studies conducted in our laboratory, the magnitude of the experimental effect (e.g., the difference between test and control groups, which is also known as the expected effect size [Glass's delta]) ranges between 1.4 and >5.0 for the primary comparisons of the mouse experiments. An anticipated effect size of 2-5, which can be detected with 79-99% power at the two-side 0.05 significance level.
Data exclusions	Data/samples were only excluded if known errors occurred during processing of samples
Replication	All ELISAs and neutrophil recruitment assays were conducted in technical and biological replicates. qPCR and Western blots were repeated in at least two biological replicates.
Randomization	Since all experiments were done with the same cell lines, no randomization was required. All cells were exposed to all treatments outlined in the study.
Blinding	Since all experiments were done with the same cell lines, no investigator blinding was required. All cells were exposed to all treatments outlined in the study.

Reporting for specific materials, systems and methods

We require information from authors about some types of materials, experimental systems and methods used in many studies. Here, indicate whether each material, system or method listed is relevant to your study. If you are not sure if a list item applies to your research, read the appropriate section before selecting a response.

Materials & experimental systems

n/a	Involved in the study	
☐	☒	Antibodies
☐	☒	Eukaryotic cell lines
☒	☐	Palaeontology and archaeology
☐	☒	Animals and other organisms
☒	☐	Clinical data
☒	☐	Dual use research of concern
☒	☐	Plants

Methods

n/a	Involved in the study	
☒	☐	ChIP-seq
☐	☒	Flow cytometry
☒	☐	MRI-based neuroimaging

Antibodies

Antibodies used	CD16/CD32 (Fc Block) Recombinant Rabbit Monoclonal Antibody (Invitrogen, Cat #14-0161-85, Lot #2493164, 1:100, clone 93); Zombie Violet Fixable Viability Kit (Biolegend, Cat #423113, Lot #B348690, 1:250); Alexa Fluor® 488 Anti-Mouse/Human CD11b Antibody (Biolegend, Cat #10217, Lot #B307666, 1:2000, clone M1/70); APC/Cyanine7 Anti-Mouse CD45 Recombinant Antibody (Biolegend, Cat #157618, Lot #B387654, 1:2000, clone QA17A26); Alexa Fluor® 647 Anti-Mouse F4/80 Antibody (Biolegend, Cat #123122, Lot #B348175, 1:1000, clone BM8); PE anti-mouse CD170 (Siglec-F) Antibody (Biolegend, Cat #155506, Lot # B357623, 1:200, clone S17007L); Brilliant Violet 650™ anti-mouse Ly-6G/Ly-6C (Gr-1) Antibody (Biolegend, Cat #108442, Lot # B391687, 1:2000, clone RB6-8C5); Monoclonal IL-8 (E5F5Q) XP Rabbit mAb (Cell Signaling Technologies, Cat #94407, Lot #08312-2f7; 1:1000); Polyclonal Swine anti-rabbit Immunoglobulin HRP conjugated (Dako, Cat #P3099, Lot #20030309, 1:1000 or 1:5000); Recombinant monoclonal anti-human GAPDH (D16H11) XP Rabbit mAb (Cell Signaling Technologies, Cat #5174, RRID:AB_10622025, 1:2000); Anti-actin mAb IgG2b (Cytoskeleton, Cat#AAN02-S, Lot #019, 1:1000, Clone 7A8.2.1); Phalloidin-iFluor™ 488 Conjugate (Cayman Chemicals, Cat #20549, Lot 0673195-1, 1:1000).
Validation	Manufacturer instructions were followed for validation and use of all antibodies. Anti-MelLec antibodies were generated and validated as follows. Sprague Dawley rats were immunised with Fc-MelLec in Freund's complete adjuvant. After a final intraperitoneal boost, without adjuvant, rat splenocytes were harvested and fused with Y3 myeloma cells, as described. Hybridoma supernatants were screened by ELISA and positives were then tested by immunohistochemistry and flow cytometry, as described below, against Fc-MelLec as well as MelLec transduced NIH3T3 fibroblasts.

Eukaryotic cell lines

Policy information about cell lines and Sex and Gender in Research

Cell line source(s)	NCI-H292 from American Type Tissue Collection (ATCC)
Authentication	The cells were not authenticated after receipt.
Mycoplasma contamination	Cell lines were not tested for mycoplasma contamination
Commonly misidentified lines (See ICLAC register)	No commonly misidentified cell lines were used in the study.

Animals and other research organisms

Policy information about studies involving animals; ARRIVE guidelines recommended for reporting animal research, and Sex and Gender in Research

Laboratory animals	6-8 week old WT C57BL/6 (JAX #000664) were used.
Wild animals	Not used
Reporting on sex	Experiments were performed in male and female mice and no differences were observed.
Field-collected samples	N/A
Ethics oversight	Massachusetts General Hospital Institutional Animal Care and Use Committee

Note that full information on the approval of the study protocol must also be provided in the manuscript.

Flow Cytometry

Plots

Confirm that:

☒ The axis labels state the marker and fluorochrome used (e.g. CD4-FITC).

☒ The axis scales are clearly visible. Include numbers along axes only for bottom left plot of group (a 'group' is an analysis of identical markers).

☒ All plots are contour plots with outliers or pseudocolor plots.

☒ A numerical value for number of cells or percentage (with statistics) is provided.

Methodology

Sample preparation	Flow cytometry was performed on BAL lavage fluid obtained from mice. Cells were pelleted, stained for live/dead staining using zombie violet viability dye, blocked using Fc block and then labelled with CD605, CD11b and Ly6-G
Instrument	BD FACSCelesta
Software	DIVA software was used for data collection and Flow Jo was used for analysis
Cell population abundance	Cells were not sorted
Gating strategy	Samples were gated using FSC-A vs SSC-A to eliminate debris, gate on cells, and gate on counting beads. Cells were then gates for single cells using FSC-A vs FSC-H followed by SSC-H vs SSC-A. Single cells populations were then gated for viability (Zombie Violet dye; BV-421) vs CD45+ cells- BV-605. Live CD45+ cells (BV421-; BV605+) were then gated for neutrophils using CD11b-APC-FIRE vs Ly6G-AF488. Neutrophils were defined as CD11b+, Ly6G+.

☒ Tick this box to confirm that a figure exemplifying the gating strategy is provided in the Supplementary Information.
